# Predictors of successful separation from high-flow nasal oxygen therapy in patients with acute respiratory failure: a retrospective monocenter study

**DOI:** 10.1186/s13613-019-0578-8

**Published:** 2019-09-11

**Authors:** Maeva Rodriguez, Arnaud W. Thille, Florence Boissier, Anne Veinstein, Delphine Chatellier, René Robert, Sylvain Le Pape, Jean-Pierre Frat, Remi Coudroy

**Affiliations:** 1 Médecine Intensive et Réanimation, CHU de Poitiers, 2 rue de la Milétrie, 86000 Poitiers, France; 2 INSERM CIC 1402, ALIVE Group, Université de Poitiers, Poitiers, France

**Keywords:** High-flow nasal oxygen therapy, Ventilator weaning, Intensive-care units, Length of stay, Observational study, Oximetry

## Abstract

**Background:**

High-flow nasal oxygen therapy (HFOT) is a promising first-line therapy for acute respiratory failure. However, its weaning has never been investigated and could lead to unnecessary prolonged intensive-care unit (ICU) stay. The aim of this study is to assess predictors of successful separation from HFOT in critically ill patients. We performed a retrospective monocenter observational study over a 2-year period including all patients treated with HFOT for acute respiratory failure in the ICU. Those who died or were intubated without prior HFOT separation attempt, who were treated with non-invasive ventilation at the time of HFOT separation, or who received HFOT as a preventive treatment during the post-extubation period were excluded.

**Results:**

From the 190 patients analyzed, 168 (88%) were successfully separated from HFOT at the first attempt. Patients who failed separation from HFOT at the first attempt had longer ICU length of stay than those who succeeded: 10 days (7–12) vs. 5 (4–8), *p* < 0.0001. Fraction of inspired oxygen (FiO_2_) ≤ 40% and a respiratory rate-oxygenation (ROX) index (calculated as the ratio of SpO_2_/FiO_2_ to the respiratory rate) ≥ 9.2 predicted successful separation from HFOT with sensitivity of 85% and 84%, respectively.

**Conclusions:**

FiO_2_ ≤ 40% and ROX index ≥ 9.2 were two predictors of successful separation from HFOT at the bedside. Prospective multicenter studies are needed to confirm these results.

## Background

High-flow nasal oxygen therapy (HFOT) is increasingly used in the management of acute respiratory failure [1]. As compared to standard oxygen, it enables improved oxygenation, alveolar ventilation, comfort, and decreased work of breathing, in hypoxemic as well as in hypercapnic respiratory failure [2, 3]. Several large randomized-controlled trials have shown similar or better outcomes with HFOT than with standard oxygen therapy [4, 5] or with non-invasive ventilation [6, 7] in preventing or treating acute respiratory failure in intensive-care unit (ICU). However, during the recovery phase of acute respiratory failure, factors associated with successful separation from HFOT have never been assessed, i.e., the bridge from HFOT to standard oxygen therapy. As the continuation of HFOT might lead to unnecessarily prolonged ICU stay, we aimed at identifying predictors of successful HFOT separation.

## Methods

We performed a retrospective observational monocenter study over a 2-year period (from January, 1st 2016 to December, 31th 2017). The study was approved by the local ethics committee, and given its non-interventional nature, informed consent was waived (CHU86-R2018-10-07).

Weaning from HFOT encompasses the reduction of FiO_2_ and flow to achieve a minimal support, and the separation attempt from HFOT per se. Given the design of the study, the reduction of HFOT support could not be assessed. Therefore, our primary outcome was to identify clinical variables associated with successful separation from HFOT.

All patients treated with HFOT for acute respiratory failure were included. Patients who died or were intubated without prior HFOT separation attempt, those who were treated with non-invasive ventilation at the time of HFOT separation, or who received HFOT as a preventive treatment during the post-extubation period (defined as the use of HFOT immediately after extubation for less than 48 h in the absence of clinical sign of respiratory failure) were excluded.

All patients were monitored until intubation, death, or successful separation from HFOT defined as discontinuation of HFOT for more than 48 h or until ICU discharge. In our unit, HFOT weaning was supervised by the attending physician and its separation usually considered when SpO_2_ was ≥ 92% with FiO_2_ ≤ 60%, independently from gas flow. HFOT separation failure was defined by respiratory failure requiring HFOT resumption, NIV initiation, intubation, or death within the first 48 h after switch from HFOT to standard oxygen.

Demographic data, ventilatory settings, and vital parameters under HFOT were collected before each separation attempt, and then under standard oxygen therapy after the separation from HFOT. For patients who failed at the first separation attempt from HFOT, these data were collected after HFOT resumption. Comorbidities, such as chronic cardiac failure (including coronary artery disease, severe valvulopathy, chronic atrial fibrillation, and heart failure of any cause) [8], chronic respiratory failure (defined as pulmonary function test alterations), and immunosuppression [9] were collected. The pulse oximetry to fraction of inspired oxygen ratio (SpO_2_/FiO_2_) and the respiratory rate-oxygenation (ROX) index (SpO_2_/FiO_2_ to respiratory rate) were calculated under HFOT before each separation attempt [10]. When available, last arterial blood gases under HFOT before each separation attempt were collected.

Continuous variables were expressed in mean ± standard deviation or median (interquartile range) according to their distribution and compared using the Mann–Whitney or the *t* test as appropriate. Categorical variables were expressed in number (percentage) and compared using the Fisher’s or Chi-square test as appropriate. Univariate analysis was performed to identify variables associated with successful separation from HFOT. For continuous variables, receiver-operating curves were plotted and the best threshold associated with successful separation from HFOT was assessed using the Youden’s index. A *p* value < 0.05 was considered significant.

## Results

**Fig. 1:**
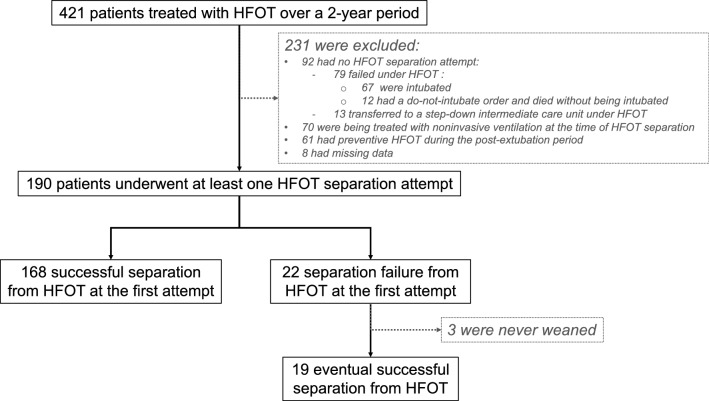
Flow chart of patients included over the study period

Among the 1494 patients admitted to our ICU over the study period, 421 were treated with HFOT. Of them, 231 were excluded for the following reasons: 92 patients had never undergone a separation attempt from HFOT (79 failed HFOT and 13 were transferred to a step-down intermediate-care unit under HFOT), 70 were under non-invasive ventilation while separation from HFOT, 61 received HFOT as a preventive treatment during the post-extubation period, and 8 had missing data (Fig. 1). As illustrated in Table 1, the 79 patients who failed HFOT (i.e., who were intubated after HFOT failure or who died after a do-not-intubate order) were older and had higher severity score upon ICU admission than the 190 who underwent at least one HFOT separation attempt.

**Table 1:** Baseline characteristics of patients who failed under HFOT and who had at least one HFOT separation attempt

Baseline characteristics	HFOT failure (*n* = 79)	At least one HFOT separation attempt (*n* = 190)	*p* value
Age, years	65 ± 12	61 ± 15	0.03
Gender, female, *n*	27 (34%)	61 (32%)	0.85
Simplified acute physiology score II, points	53 ± 21	37 ± 12	< 0.001
Sequential organ failure assessment score, points	8 ± 5	5 ± 3	< 0.001

*HFOT* high-flow nasal oxygen therapy

At the first attempt, 168 out of the 190 patients (88%) retained in the analysis were successfully separated from HFOT, whereas 22 (12%) failed. Their characteristics are displayed in Table 2. Comorbidities were not different according to the outcome of the first HFOT separation attempt. Likewise, main reason for acute respiratory failure was not different according to the outcome of the first HFOT separation attempt, except for a higher proportion of pneumonia in the failed than in the successful HFOT separation group. Maximal gas flow and FiO_2_ set under HFOT were 50 ± 3 L/min and 75 ± 21%, respectively, and did not differ between the two groups (Table 2).

**Table 2:** Characteristics of patients according to the success or failure of the first separation attempt from high-flow nasal oxygen therapy

Variables	Success (*n* = 168)	Failure (*n* = 22)	*p* value
Baseline characteristics			
Age, years	61 ± 15	61 ± 14	0.93
Gender, female, *n*	55 (33%)	6 (27%)	0.81
Simplified acute physiology score II, points	36 ± 12	40 ± 13	0.22
Sequential organ failure assessment score, points	5 ± 3	5 ± 4	0.74
Comorbidities, *n*			
Chronic cardiac failure	32 (19%)	4 (18%)	> 0.99
Chronic respiratory failure			
Obstructive	31 (18%)	5 (23%)	0.85
Restrictive	10 (5.9%)	2 (9.1%)	0.92
Immunosuppression	56 (33%)	8 (36%)	0.97
Main reason for acute respiratory failure, *n*			
Pneumonia	79 (47%)	16 (73%)	0.04
Cardiogenic pulmonary edema	28 (17%)	1 (4.5%)	0.21
Extrapulmonary sepsis	26 (15%)	1 (4.5%)	0.32
Acute exacerbation of chronic respiratory failure	9 (5.4%)	1 (4.5%)	> 0.99
Pulmonary embolism	7 (4.2%)	1 (4.5%)	> 0.99
Shock	4 (2.4%)	0 (0%)	> 0.99
Neurological failure	4 (2.4%)	0 (0%)	> 0.99
Drug abuse	2 (1.2%)	0 (0%)	> 0.99
Other diagnoses	1 (6.0%)	2 (9.1%)	0.63
Characteristics before the first separation attempt from HFOT			
Bilateral infiltrates at chest X-ray, *n*	59 (35%)	10 (45%)	0.48
Vasoactive drugs, *n*	8 (5%)	0 (0%)	0.63
Length of treatment with HFOT, hours	41 (23–72)	56 (39–80)	0.26
Last clinical and biological parameters before the first separation attempt from HFOT			
Flow, L/min	42 ± 8	43 ± 8	0.54
FiO_2_ set, %	39 ± 7	48 ± 16	0.02
Temperature, °C	37.3 ± 0.8	37.4 ± 0.8	0.54
Systolic arterial pressure, mmHg	125 ± 19	127 ± 19	0.68
Heart rate, beats/min	92 ± 19	94 ± 20	0.60
Respiratory rate, breaths/min	21 ± 5	22 ± 5	0.59
Pulse oximetry, %	96 ± 2	96 ± 2	0.36
SpO_2_/FiO_2_, %	254 ± 45	217 ± 53	0.004
ROX index	12.7 ± 1.2	10.2 ± 3.0	0.002
PaO_2_, mmHg	95 ± 33	96 ± 33	0.92
PaCO_2_, mmHg	35 ± 7	36 ± 8	0.68
pH	7.45 ± 0.05	7.44 ± 0.05	0.72
Serum bicarbonate, mmol/L	24 ± 5	24 ± 5	0.99
Last PaO_2_/FiO_2_ under HFOT, mmHg	226 ± 64	210 ± 72	0.35
Sequential organ failure assessment score, points	3 ± 2	1 ± 2	0.27
First clinical parameters under standard oxygen after the separation attempt from HFOT			
Flow, L/min	4 ± 1	5 ± 1	0.02
Temperature, °C	37.4 ± 0.7	37.4 ± 0.9	0.92
Systolic arterial pressure, mmHg	125 ± 18	131 ± 17	0.15
Heart rate, beats/min	91 ± 18	92 ± 16	0.75
Respiratory rate, breaths/min	23 ± 5	22 ± 5	0.39
Pulse oximetry, %	96 ± 3	95 ± 3	0.13
SpO_2_/FiO_2_, %	286 ± 39	263 ± 28	0.001
ROX index	13.3 ± 3.9	12.7 ± 3.2	0.47
Outcomes			
Total length of HFOT treatment, h	41 (23–72)	84 (61–118)	< 0.0001
ICU length of stay, days	5 (4–8)	10 (7–12)	< 0.0001
Time from HFOT separation success to ICU discharge, days	2 (1–3)	2 (2–3)	0.21

*HFOT* high-flow nasal oxygen therapy, *ICU* intensive-care unit, *FiO*_*2*_ fraction of inspired oxygen, *PaO*_*2*_ partial pressure in oxygen, *SpO*_*2*_ oxygen saturation, *ROX* SpO_2_/FiO_2_ to respiratory rate

**Fig. 2:**
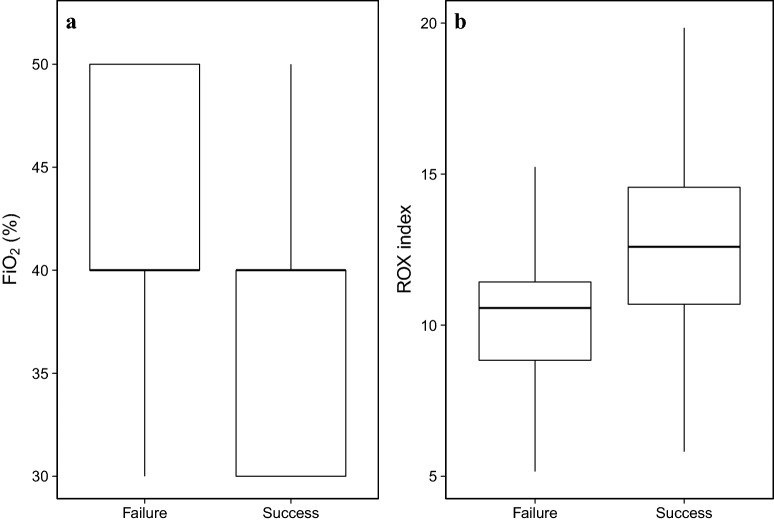
Last physiological parameters recorded at the bedside under high-flow nasal oxygen therapy before the first separation attempt. **a** Box-plot of FiO_2_ according to the success or failure of the first separation attempt (*p* = 0.02). **b** Box-plot of the ROX index according to the success or failure of the first separation attempt (*p* = 0.002)

Patients who were successfully separated from HFOT at the first attempt required less oxygen and were more likely to have FiO_2_ ≤ 40% (85% vs. 59%, *p* = 0.007, Table 2 and Fig. 2) than those who failed. A FiO_2_ ≤ 40% predicted successful separation from HFOT at the first attempt with sensitivity of 85%, specificity of 41%, positive predictive value of 92%, negative predictive value of 26%, and accuracy of 80%. Likewise, the ROX index was higher in patients who were successfully separated from HFOT at the first attempt than in those who failed (12.7 vs. 10.2, *p* = 0.002) (Table 2 and Fig. 2). An ROX index ≥ 9.2 predicted successful separation from HFOT at the first attempt with sensitivity of 84%, specificity of 50%, positive predictive value of 93%, negative predictive value of 30%, and accuracy of 80%.

ICU length of stay was significantly longer among patients who failed separation from HFOT at the first attempt than in those who succeeded: 10 days [7–12] vs. 5 [4–8], respectively (*p* < 0.0001). HFOT was resumed after 13 h [5–26] under standard oxygen. The main reason for HFOT separation failure at the first attempt was hypoxemia in 95% of cases (21/22). After HFOT resumption, SpO_2_/FiO_2_, ROX index, and PaO_2_/FiO_2_ under HFOT were lower than the last values measured under HFOT before the first separation attempt (Table 3). Interestingly, HFOT was resumed at higher flow than before the first separation attempt, whereas FiO_2_ was comparable between the two time periods. There was no difference in the last characteristics under HFOT before the first separation attempt according to the main reason for respiratory failure (Additional file 1: Table S1). After separation failure at the first attempt, 86% (19 out of 22) of patients were eventually separated successfully from HFOT. Their characteristics did not change between the HFOT separation success and the prior HFOT separation failure, except for higher ROX index at the time of the separation success than the separation failure, 12.4 ± 3.8 vs. 10.1 ± 3.1, *p* = 0.04.

**Table 3:** Clinical and biological parameters under high-flow nasal oxygen therapy in the failure group, before separation and after failure

	Last values under HFOT before HFOT separation	First values under HFOT after HFOT resumption	*p* value
Temperature, °C	37.4 ± 0.8	37.4 ± 0.8	0.89
Respiratory rate, /min	22 ± 5	21 ± 5	0.52
Pulse oximetry, %	96 ± 2	96 ± 3	0.89
Systolic arterial pressure, mmHg	127 ± 19	131 ± 17	0.32
Heart rate, /min	94 ± 20	97 ± 20	0.28
Flow, L/min	43 ± 8	50 ± 5	0.001
FiO_2_, %	48 ± 16	56 ± 15	0.12
SpO_2_/FiO_2_, %	217 ± 53	183 ± 41	0.03
ROX index	10.2 ± 3.0	9.1 ± 3.7	0.30
PaO_2_, mmHg	96 ± 33	*n* = 16 84 ± 20	0.09
PaCO_2_, mmHg	36 ± 8	*n* = 16 43 ± 20	0.18
pH, mmHg	7.44 ± 0.05	*n* = 16 7.42 ± 0.10	0.52
Serum bicarbonate, mmol/L	24 ± 5	*n* = 16 26 ± 5	0.08
PaO_2_/FiO_2_ under HFOT, mmHg	210 ± 72	*n* = 16 148 ± 38	0.006

*HFOT* high-flow nasal oxygen therapy, *ICU* intensive-care unit, *FiO*_*2*_ fraction of inspired oxygen, *PaO*_*2*_ partial pressure in oxygen, *SpO*_*2*_ oxygen saturation, *ROX* SpO_2_/FiO_2_ to respiratory rate

## Discussion

This study assessed predictors of successful separation from HFOT initiated for management of acute respiratory failure in the ICU. Our main findings are that only two bedside criteria, namely FiO_2_ and the ROX index calculated under HFOT, were different between patients who failed and those who succeeded in the first HFOT separation attempt.

FiO_2_ ≤ 40% was a predictor of separation success from HFOT with sensitivity reaching 85%, whereas this was not the case for HFOT flow. Therefore, this finding might suggest hastening the reduction of FiO_2_ rather than flow when weaning from HFOT. It is interesting to note that weaning from mechanical ventilation is recommended starting from a FiO_2_ ≤ 40%, illustrating the importance of decreasing oxygen requirements before separation from oxygenation support. Moreover, FiO_2_ of 45% is theoretically achievable under standard oxygen therapy with a flow of 5 L/min as a bridge to HFOT separation [11]. Therefore, separation from HFOT is more likely to be successful if a same range of FiO_2_ can be reached under standard oxygen therapy than under HFOT.

The ROX index was higher in patients successfully separated from HFOT. This score was initially built to predict the success of HFOT in patients with severe pneumonia with more accuracy than each of its components independently [10]. The best ROX index threshold predicting successful separation of HFOT was 9.2 using the Youden index. However, this threshold had poor specificity of only 50%, suggesting that half of patients who failed had ROX index higher than 9.2. To limit the risk related to HFOT separation failure, we identified the best positive predictive value for the ROX index.

Patients who failed the first HFOT separation attempt had lower oxygenation indices (SpO_2_/FiO_2_, ROX index, and PaO_2_/FiO_2_) after HFOT resumption than before HFOT separation, whereas FiO_2_ was not different between the two time periods. This difference in oxygenation could have been explained by HFOT flow-dependent lung recruitment. Indeed, increasing HFOT flow can increase end-expiratory lung volume and consequently improve oxygenation without changes in FiO_2_ [12]. Whether the assessment of end-expiratory lung impedance using electrical impedance tomography (a surrogate of end-expiratory lung volume) during HFOT flow reduction could help identifying patients who will fail HFOT separation is unknown [13]. Importantly, there were no differences between patients who failed according to the main reason for respiratory failure.

Our study has some limitations. First, this is a retrospective monocenter study which may limit the external validity of our results. However, our center is an expert in this technique, as illustrated by the high case volume of patients treated with HFOT. Second, there is no recommendation for the weaning of HFOT and it is possible that the first separation attempt occurred late, explaining the low rate of separation failure observed. There is an urgent need for studies assessing the reduction of HFOT settings to reach minimal support. Indeed, whether a reduction of FiO_2_ or flow would be the most suitable option to reach minimal support before the separation attempt is unknown and could not be tested given the design of our study. Finally, the clinical relevance of separation failure from HFOT is debatable. Indeed, its consequences are probably less severe than weaning failure from invasive ventilation, which is associated with a twofold increase in mortality [14]. However, patients who failed at the first attempt of separation from HFOT had significantly longer ICU stay than those who succeeded. Moreover, HFOT usually requires close monitoring in ICU or in intermediate-care unit, which represents a non-negligible cost. The economic impact of a weaning protocol is likely to be considered in further studies.

## Conclusion

In this retrospective monocenter study, predictors of successful separation from HFOT at the bedside were FiO_2_ ≤ 40% and ROX index ≥ 9.2. The most interesting indicator might be FiO_2_, since its performance is similar to the ROX index and does not require any calculation at the bedside. A multicenter prospective study is mandatory to confirm the usefulness of these two easy-to-assess parameters with the aim of predicting successful separation from HFOT. Moreover, studies are needed to assess the most efficient way to reach minimal HFOT support.

## Supplementary information

**Additional file 1: Table S1.** Clinical and biological parameters collected under high-flow nasal oxygen therapy before the first separation attempt according to the reason for acute respiratory failure among the 22 patients who failed the separation attempt.

## Data Availability

All data generated or analyzed during this study are included in this published article [and its additional information files].

## Abbreviations

HFOT: high-flow nasal oxygen therapy
ICU: intensive-care unit
FiO_2_: fraction of inspired oxygen
SpO_2_: oxygen saturation
ROX: respiratory rate oxygenation
SAPS II: simplified acute physiology score II
SOFA: sequential organ failure assessment
