# Supplementary material for: Predictors of successful separation from high-flow nasal oxygen therapy in patients with acute respiratory failure: a retrospective monocenter study
Source: Ann Intensive Care. 2019 Sep 11;9:101. doi: 10.1186/s13613-019-0578-8 (PMC6738360; doi:10.1186/s13613-019-0578-8)
Supplement: Supplementary file 1 — Additional file 1: Table S1. Clinical and biological parameters collected under high-flow nasal oxygen therapy before the first separation attempt according to the reason for acute respiratory failure among the 22 patients who failed the separation attempt. [file 13613_2019_578_MOESM1_ESM.docx]

**Additional file 1: Table S1. Clinical and biological parameters collected under high-flow nasal cannula oxygen therapy before the first separation attempt according to the reason for acute respiratory failure among the 22 patients who failed the separation attempt.**

|  | **Pneumonia**  **(n=16)** | **Other reason than pneumonia (n=6)** | **P value** |
| --- | --- | --- | --- |
| Time of failure, hours | 13 (4-28) | 12 (6-21) | 0.74 |
| Temperature, °C | 37.3 (36.8-37.7) | 37.7 (37.0-38.3) | 0.24 |
| Respiratory rate, /min | 21 (21-25) | 21 (19-22) | 0.43 |
| Pulse oximetry, % | 96 (84-97) | 98 (94-98) | 0.48 |
| Systolic arterial pressure, mm Hg | 118 (110-140) | 136 (126-145) | 0.30 |
| Heart rate, /min | 93 (79-108) | 91 (86-102) | 0.80 |
| Flow, l/min | 40 (40-50) | 50 (43-50) | 0.25 |
| FiO_2_, % | 40 (40-50) | 40 (40-70) | >0.99 |
| SpO_2_/FiO_2_, % | 235 (192-241) | 238 (151-244) | 0.98 |
| ROX index | 9.1 (8.8-11.4) | 10.8 (9.3-11.9) | 0.92 |
| PaO_2_, mm Hg | 85 (67-102) | 105 (96-123) | 0.36 |
| PaCO_2_, mm Hg | 37 (34-42) | 32 (31-35) | 0.35 |
| pH | 7.44 (7.44-7.48) | 7.43 (7.41-7.46) | 0.50 |
| Serum bicarbonate, mmol/l | 24 (22-26) | 22 (20-25) | 0.53 |
| PaO_2_/FiO_2_ under HFOT, mm Hg | 188 (161-219) | 251 (194-315) | 0.10 |

HFOT: High-flow nasal cannula oxygen therapy, ICU: intensive care unit, FiO_2_: fraction of inspired oxygen, PaO_2_: Partial pressure in oxygen, SpO_2_: Oxygen saturation, ROX: SpO_2_/FiO_2_ to respiratory rate.
